# Supplementary material for: Spatial modeling connecting childhood atopic dermatitis prevalence with household exposure to pollutants
Source: Commun Med (Lond). 2024 Apr 18;4:74. doi: 10.1038/s43856-024-00500-3 (PMC11026442; doi:10.1038/s43856-024-00500-3)
Supplement: Supplementary file 2 — Supplementary Information [file 43856_2024_500_MOESM2_ESM.pdf]

# Spatial modeling connecting childhood atopic dermatitis prevalence with household exposure to pollutants

Grace Ratley[1,+], Jordan Zeldin[1,+], Ashleigh A Sun[1], Manoj Yadav[1], Prem Prashant Chaudhary[1], and Ian A. Myles[1,*]

1 – Epithelial Therapeutics Unit, National Institute of Allergy and Infectious Disease, National Institutes of Health, Bethesda, MD, USA

+Denotes equal contribution.

*Corresponding author: mylesi@niaid.nih.gov.
9000 Rockville Pike
Building 29B, Room 5G17
Bethesda, MD 20893

# Supplemental Materials

Supplemental Fig 1

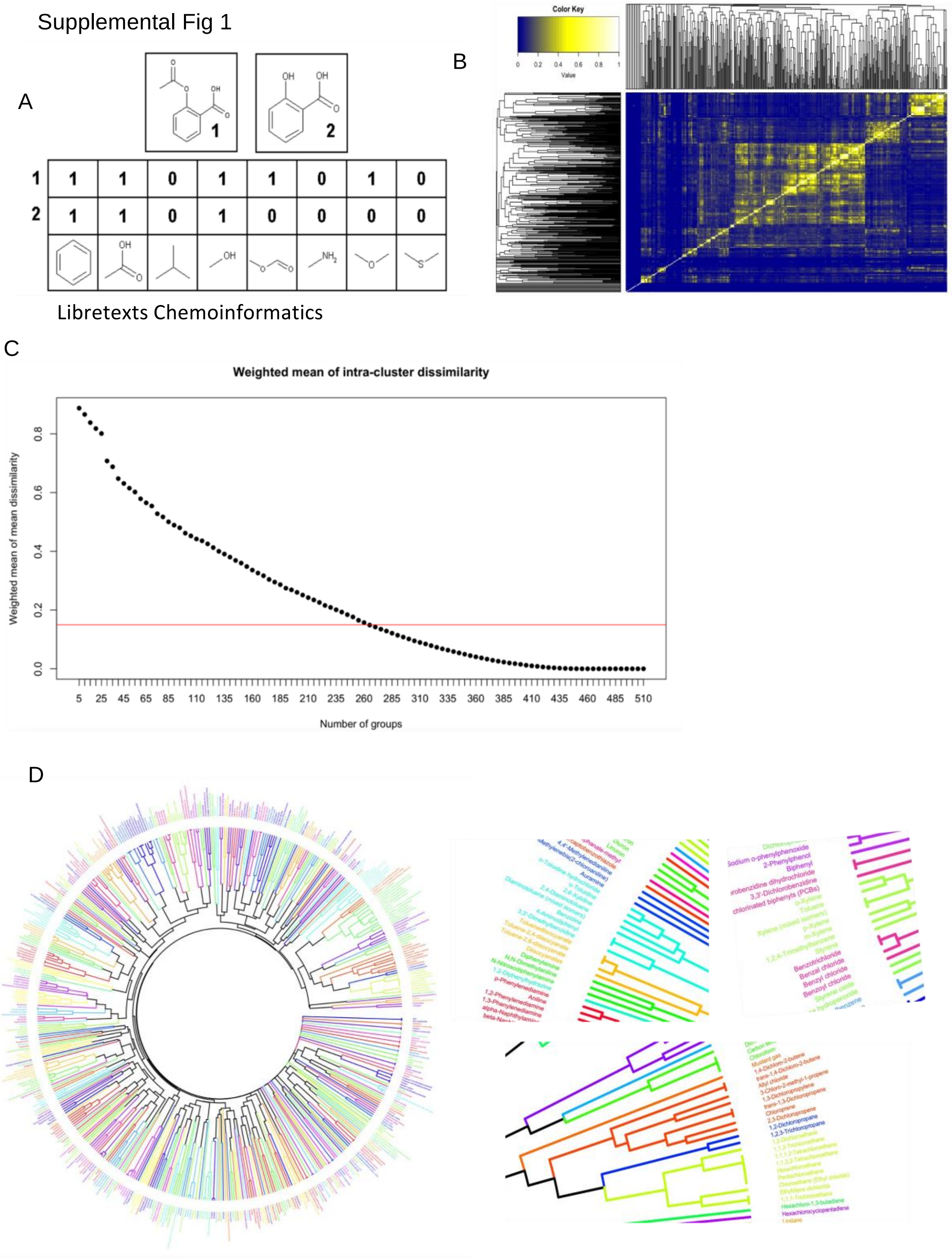

**Supplemental Figure 1. Clustering of chemical structure and spatial distribution assumptions.** (A) Example of breaking down of two molecules into binary fingerprints from Libretexts Chemoinformatics. (B) Similarity matrix using Tanimoto distance of all molecules, demonstrating the range of hierarchical clustering. Document that translates between molecular similarity clusters and their component compounds is provided separately. (C) the relationship between the number of groups in the hierarchical cluster and the average amount of dissimilarity within the groups, the red line indicating the threshold of 0.85 after which we assume there are diminishing returns to smaller groups. (D) All of the chemicals common to all three years in a hierarchical cluster, color-coded by the optimal threshold determined in part B, zoomed in segments for readability.

Supplemental Fig 2

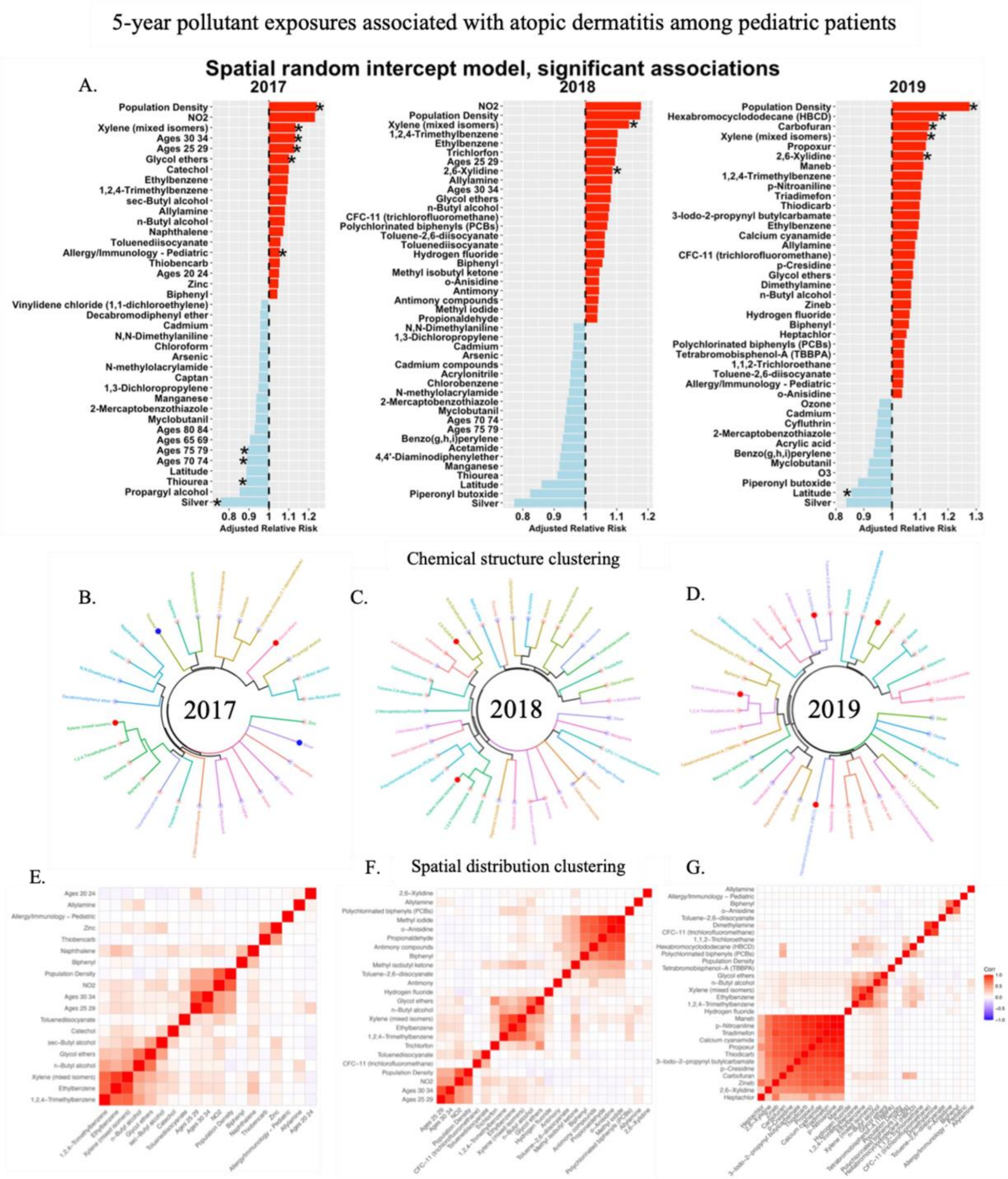


**Supplemental Figure 2. Results of mixed effect model with chemical and geographic clusters.** (A) Adjusted relative risk of pollution concentration of visitation rate for atopic dermatitis from

negative binomial model with nested random intercept structure for spatial effects. All compounds represented are statistically significant to a p-value threshold of 0.05, but only those with asterisk were significant after Benjamini-Hochberg adjustment for false discover rate. (B-D) For 2017-2019, the molecular similarity clusters of those compounds that were significant from A. Those that are opaque are significant after Benjamini-Hochberg adjustment. Red indicates positive association and blue negative. Color codes of the chemical clusters are based on the same algorithm described for the entire chemical data set. (E-G) For 2018-2019, correlogram of geographical association between pollution concentration, after the weighting adjustments, demonstrating which pollutants occupy similar geographical spaces and likely emitted from similar industrial processes. Spreadsheet for all results provided separately.

Supplemental Fig 3

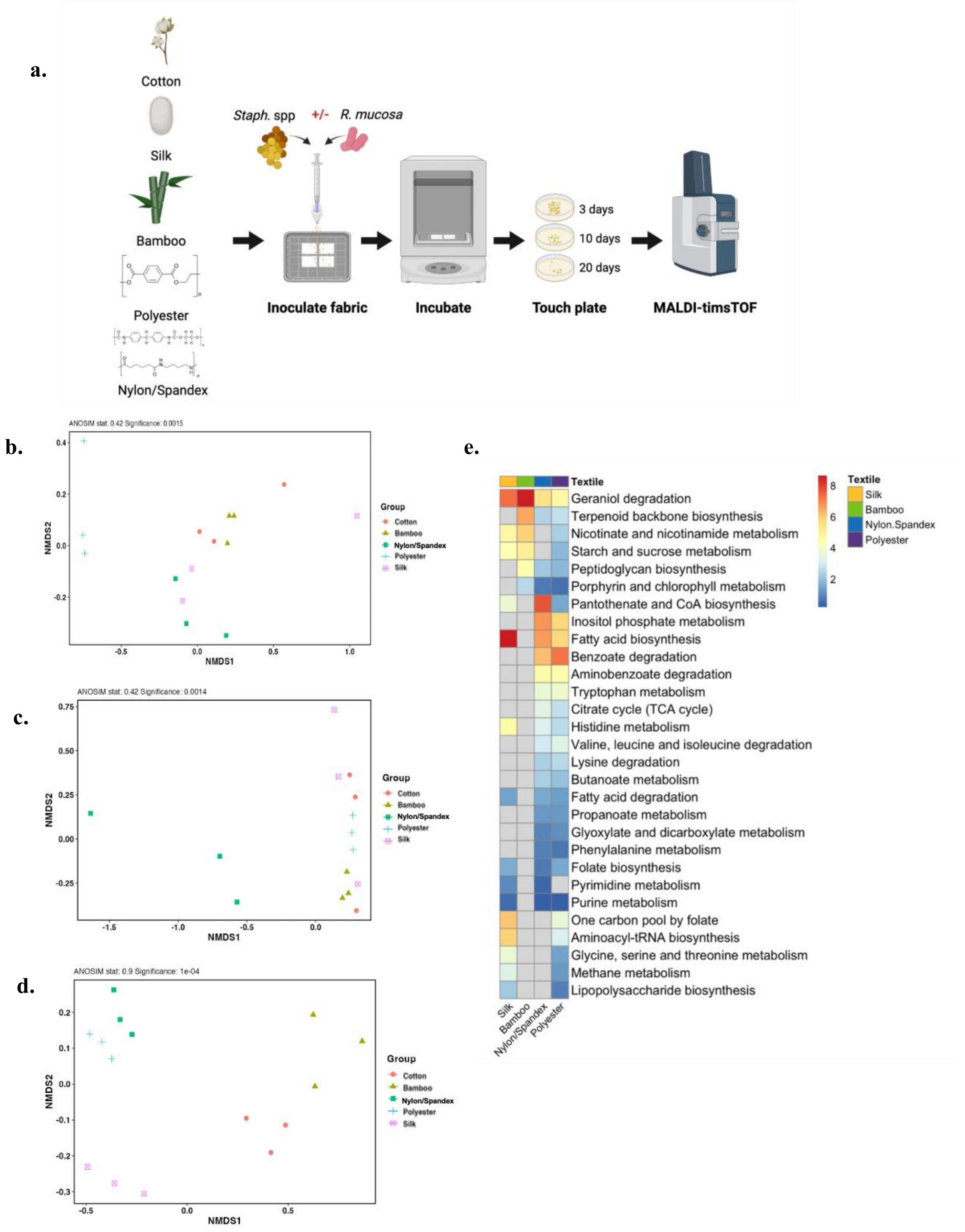


**Supplementary Figure 3.** (A) Diagram of experimental design. Fabrics were inoculated with various organisms, incubated on only the fabric for up to 20 days, and then touch-plated onto agar

for analysis after viable colonies had formed. Created with BioRender.com (B-C) NMDS plot for metabolomics of *R. mucosa* cultured on indicated fabrics for 3 days (B) or 20 days (C), (D) NMDS plot for *S. epidermidis* cultured on indicated fabrics for 3 days. (E) Heatmap of IPS values from Day 20 comparing *S. epidermidis* metabolism grown on various textiles to growth on cotton. Pathways with 2 or more values across samples were selected. Results represent three or more independent experiments.

Supplemental Fig 4

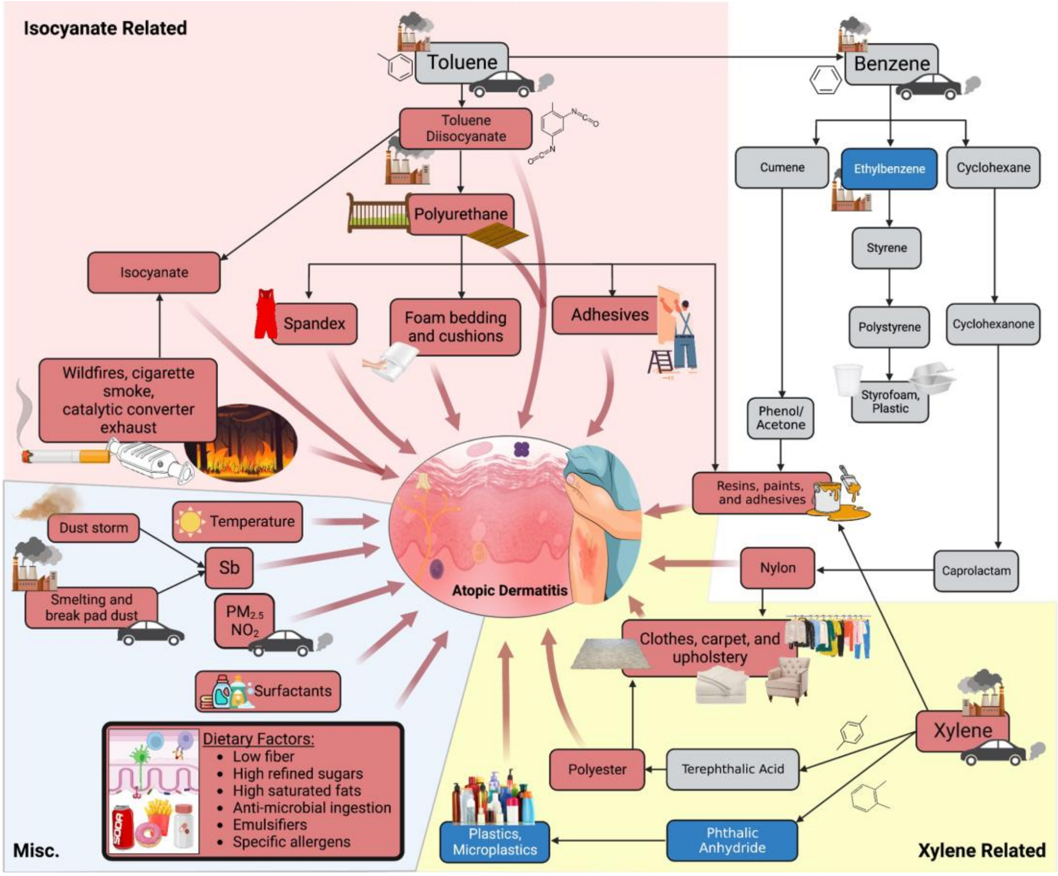


**Supplementary Figure 4.** Overall summary of current hypothesis linking environmental exposures to atopic dermatitis pathology. Created using BioRender.com with additional images purchased from iStock.com.

**Supplementary Note 1**
**Survey text:**

**Between the time of conception and YOUR CHILD'S 3rd birthday, did you make any of the following changes to your home? Check all that apply:**

- Brought in a new foam mattress
- Brought in new furniture
- Brought in a new refrigerator, stove, or dishwasher
- Brought in anything you knew had flame retardant applied
- Re-sealed or replaced hardwood floors
- Replaced carpeting
- Painted the walls
- Added, replaced, or removed wallpaper
- Used polyester sheets on their bed
- At least one person in the home smoked cigarettes (even if only outdoors)
- New landscaping outdoors
- Had a cat in the home
- Had a dog in the home
- None of the above
- I don't know/remember
